# Immunoreactivity of the *Mycobacterium avium *subsp. *paratuberculosis *19-kDa lipoprotein

**DOI:** 10.1186/1471-2180-5-3

**Published:** 2005-01-21

**Authors:** Jason FJ Huntley, Judith R Stabel, John P Bannantine

**Affiliations:** 1 Department of Microbiology, University of Texas Southwestern Medical Center, Dallas, TX 75390-9048, USA; 2 Bacterial Diseases of Livestock Research Unit, National Animal Disease Center-ARS-USDA, 2300 Dayton Avenue, Ames, IA 50010, USA

## Abstract

**Background:**

The *Mycobacterium tuberculosis *19-kDa lipoprotein has been reported to stimulate both T and B cell responses as well as induce a number of Th1 cytokines. In order to evaluate the *Mycobacterium avium *subsp. *paratuberculosis *(*M. avium *subsp. *paratuberculosis*) 19-kDa lipoprotein as an immunomodulator in cattle with Johne's disease, the gene encoding the 19-kDa protein (MAP0261c) was analyzed.

**Results:**

MAP0261c is conserved in mycobacteria, showing a 95% amino acid identity in *M. avium *subspecies *avium*, 84% in *M. intracellulare *and 76% in *M. bovis *and *M. tuberculosis*. MAP0261c was cloned, expressed, and purified as a fusion protein with the maltose-binding protein (MBP-19 kDa) in *Escherichia coli*. IFN-γ production was measured from 21 naturally infected and 9 control cattle after peripheral blood mononuclear cells (PBMCs) were stimulated with a whole cell lysate (WCL) of *M. avium *subsp. *paratuberculosis *or the recombinant MBP-19 kDa. Overall, the mean response to MBP-19 kDa was not as strong as the mean response to the WCL. By comparison, cells from control, non-infected cattle did not produce IFN-γ after stimulation with either WCL or MBP-19 kDa. To assess the humoral immune response to the 19-kDa protein, sera from cattle with clinical Johne's disease were used in immunoblot analysis. Reactivity to MBP-19 kDa protein, but not MBP alone, was observed in 9 of 14 infected cattle. Antibodies to the 19-kDa protein were not observed in 8 of 9 control cows.

**Conclusions:**

Collectively, these results demonstrate that while the 19-kDa protein from *M. avium *subsp. *paratuberculosis *stimulates a humoral immune response and weak IFN-γ production in infected cattle, the elicited responses are not strong enough to be used in a sensitive diagnostic assay.

## Background

Paratuberculosis (Johne's disease) is caused by *Mycobacterium avium *subsp. *paratuberculosis *(referred to hereafter as *M. avium *subsp. *paratuberculosis*) and induces a chronic enteritis in ruminants. The disease signs include weight loss, diarrhea, and decreased milk production. In the United States alone the economic burden of Johne's disease is estimated at over $200 million in lost annual revenue to the dairy industry [1]. Prevalence studies in the United States have estimated that between 20 to 30% of dairy herds are infected with *M. avium *subsp. *paratuberculosis *[2,3]. Neonatal calves are most susceptible to infection and are likely to become infected after ingestion of contaminated milk or colostrum [4,5]. During the subclinical stage of infection, the host cell-mediated immune response is robust and appears to control the infection. As the disease progresses from the subclinical to the clinical stage, the cell-mediated response diminishes, and a humoral immune response predominates [6]. Vaccines are not completely protective, but have been reported to reduce fecal shedding and delay the onset of clinical disease [7-9].

Lipoproteins have long been considered immunomodulators and mycobacteria are especially rich in these post-translationally modified proteins. There are approximately 100 open reading frames identified in the *M. tuberculosis *genome that possess a characteristic amino-terminal acylation motif [10]. The 19-kDa lipoprotein from *Mycobacterium tuberculosis *is immunodominant in both mice [11-13] and humans [14,15] and has been shown to stimulate CD4^+ ^T cell proliferation as well as the release of IL-2, IFN-γ, and IL-12 [16,17]. Acylation near the N-terminal portion of the 19-kDa protein is believed to occur at amino acids 19–24 and contributes to its immunogenicity [12]. Furthermore, glycosylation of the *M. tuberculosis *19-kDa protein inhibits innate immune responses, such as the release of TNF-α, IL-6, and IL-10 from macrophages, but does not affect antibody binding [18-20]. The 19-kDa protein was also shown to induce CD8+ cells to secrete IFN-γ and specifically lyse *M. tuberculosis*-infected monocytes [21] as well as promote neutrophil priming and activation [22]. Finally, B cell epitopes have been described that localize to the linear sequences of amino acids 11–30, 29–47, 61–80, and 140–159, as well as a conformation-dependent epitope at the amino and carboxy-terminal ends because of intramolecular disulfide bonding of cysteine residues [21,23]. Experimental infection and staining of macrophages has shown that the 19-kDa protein is secreted by live *M. tuberculosis *residing within the phagolysosomal compartment [24]. Homologues of the 19-kDa lipoprotein exist in *M. bovis*, *M. avium*, and *M. intracellulare *but are absent from *M. phlei*, *M. smegmatis*, *M. fortuitum*, *M. gordonae*, and *M. leprae *[25].

Despite decades of research, little is known about the *M. avium *subsp. *paratuberculosis *proteins involved in metabolism, cell wall synthesis, macrophage entry and survival, disease pathogenesis, or host immune evasion. However, several antigens have recently been identified and their immunogenicity examined by serodiagnostic and/or lymphocyte stimulation assays [26-30]. With the genome sequence of *M. avium *subsp. *paratuberculosis *recently defined [31], all proteins produced by this pathogen are now identified and can be characterized. Novel opportunities arising from the genome sequence of *M. avium *subsp. *paratuberculosis *enable us to select and characterize genes of interest. A major goal of this laboratory is to define a complete catalog of immunodominant antigens in *M. avium *subsp. *paratuberculosis*. With the immunostimulatory capabilities of the *M. tuberculosis *19-kDa antigen in mind, the objective of this study was to determine if the 19-kDa protein of *M. avium *subsp. *paratuberculosis *possessed a similar capacity. In this study, the 19-kDa lipoprotein from *M. avium subspecies paratuberculosis *was cloned, expressed, and characterized. In addition, the purified recombinant protein was used to assess cellular immune responses in subclinically infected cattle as well as humoral immune responses in cattle with clinical Johne's disease.

## Results

### Sequence analysis of the mycobacterial 19-kDa coding region

**Figure 1:**
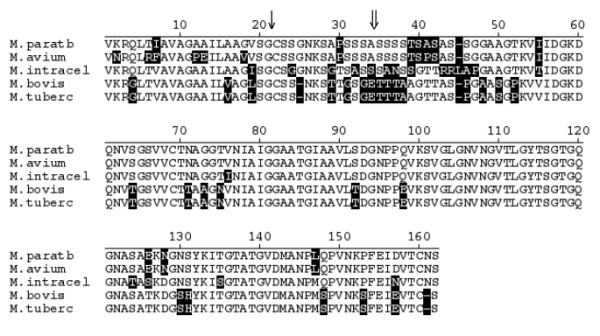
**Amino acid sequence comparison of the mycobacterial 19 kDa protein**. Non-conserved residues are shaded in black and gaps in amino acid sequence are indicated by hyphens (-). The predicted signal peptidase cleavage site for *M. tuberculosis *is indicated by a single arrow (↓), while the site for *M. avium *subsp. *paratuberculosis *is indicated by a double arrow (⇓). The GenBank accession number and abbreviations are *M. avium *subsp. *paratuberculosis *(M. paratb; AAS02578), *M. avium *subsp. *avium *(M. avium; AAB25888), *M. intracellulare *(M. intracel; AAB25885), *M. bovis *(M. bovis; S11234) and *M. tuberculosis *(M. tuberc; NP_218280).

The 19-kDa coding sequence was identified from the *M. avium *subsp. *paratuberculosis *genome project as MAP0261c. Comparison of amino acid sequences from other species of mycobacteria show that this gene product is conserved. Sequence alignment shows the N-terminal half is more variable and the region between amino acids 99 and 123 is highly conserved (Figure 1). MAP0261c displays a 95% amino acid identity in *M. avium*, 84% in *M. intracellulare *and 76% in *M. bovis *and *M. tuberculosis*. MAP0261c has a G+C content of 66.2% and encodes for 161 amino acids with a predicted molecular mass of 15.2 kDa. The first 22 amino acids of the *M. tuberculosis *19-kDa protein are hydrophobic and were previously noted to represent a signal peptide that is post-translationally cleaved to expose an N-terminal cysteine [32]. Signal peptidase cleavage analysis of MAP0261c (SignalP3.0; ) detected a signal peptide generated from a putative cleavage site between amino acids 34 and 35, to expose an N-terminal serine (Figure 1). The SignalP-NN (neural networks) model assigned the highest cleavage probability values to amino acid 35 (C score = 0.324; Y score = 0.439) with the predicted length of the signal peptide being 34 amino acids. This is slightly longer than most signal sequences which range from 18 to about 30 amino acid residues in length [33]. The predicted peptidase cleavage sight for *M. tuberculosis *is between amino acids 21 and 22 and therefore falls within this range (Figure 1). Despite a predicted signal peptidase cleavage site, PSORTb analysis software  could not predict if the protein was cytoplasmic or membrane located.

### Cloning and expression of the *M. avium *subsp. *paratuberculosis *19-kDa protein

**Figure 2:**
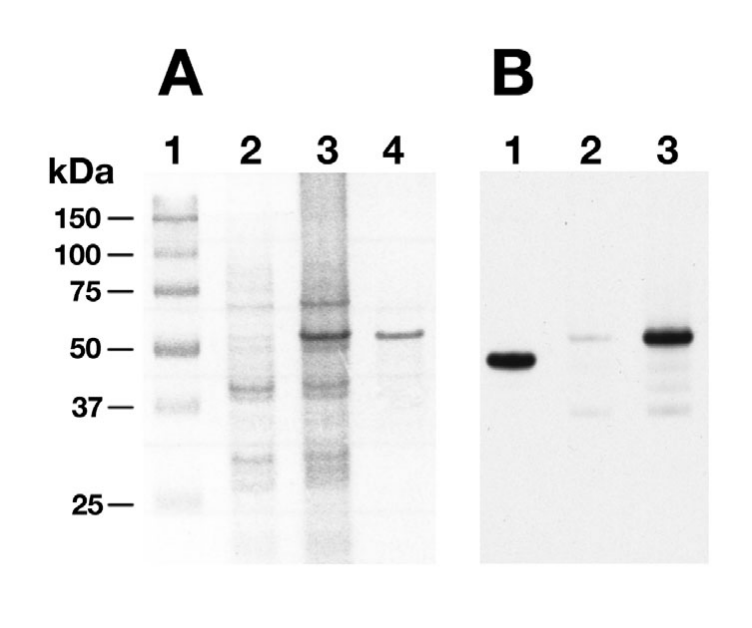
**SDS-PAGE and immunoblot analysis of the MBP-19 kDa fusion protein**. (A) An SDS-PAGE gel showing the noninduced and IPTG-induced *E. coli *protein lysates. Lanes: 1- protein size markers; 2- noninduced *E. coli *MBP-19 kDa; 3- IPTG induced *E. coli *MBP-19 kDa; 4- Affinity purified MBP-19 kDa. (B) Immunoblot probed with a monoclonal antibody to MBP. Lane 1- purified MBP; Lane 2- noninduced *E. coli *MBP-19 kDa; Lane 3- IPTG induced *E. coli *MBP-19 kDa.

In order to perform immunoassays with purified 19-kDa protein, MAP0261c was amplified from *M. avium *subsp. *paratuberculosis *genomic DNA and cloned into the pMal-c2 expression vector and transformed in *E. coli*. Induced expression resulted in production of a maltose binding protein (MBP)-19 kDa fusion protein that was affinity-purified from *E. coli *lysates. MBP-19 kDa was analyzed by SDS-PAGE (Figure 2) to assess yield, purity and size. The predicted mass of MBP alone is 42 kDa, while the predicted mass of the MBP-19 kDa fusion protein is 56 kDa. The purified MBP-19 kDa protein migrated to a position around 50 kDa in SDS-PAGE (Figure 2A). Approximately 5 mg of purified protein was easily obtained from a 500-ml broth culture at O.D._600 nm _= 0.9. The purified protein was further characterized by immunoblot analysis using a monoclonal antibody (mAb) that detects the MBP affinity tag (Figure 2B). Both the fusion protein and MBP alone are detected by the mAb. In addition, the fusion protein is expressed at higher levels under inducing conditions.

### Immunoblot analysis of the 19-kDa protein

**Figure 3:**
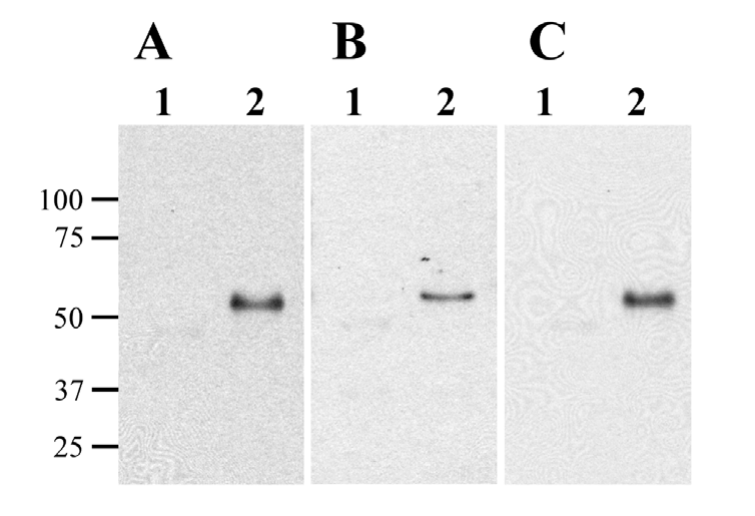
**The recombinant 19-kDa protein is recognized by serum from infected cattle**. Affinity purified MBP (lane 1) and MBP-19 kDa (lane 2) were transferred onto membranes in equal amounts and probed with sera from three clinically infected cattle: (A) animal 107; (B) animal 116; (C) animal 167. Reactivity was observed for the 19-kDa protein but not the MBP protein. Size standards are indicated in kilodaltons in the left margin.

The *M. tuberculosis *19-kDa protein is known to be immunodominant, therefore immunoblot analysis was performed to determine if cattle naturally infected with *M. avium *subsp. *paratuberculosis *produce antibodies against the 19-kDa protein. Immunoblots were probed with sera from 9 non-infected and 14 clinically infected cattle. Sera from 8 of 9 non-infected cattle did not react with either MBP or MBP-19 kDa, while 1 non-infected cattle weakly recognized both MBP and MBP-19 kDa protein (data not shown). By comparison, sera from 9 of 14 infected cattle reacted specifically with the 19-kDa protein, but not MBP alone. Antibody reactivity to the 19-kDa protein from 3 clinical cows is shown in Figure 3. Sera from the remaining five infected cattle detected both MBP and MBP-19 kDa proteins. This result made it difficult to distinguish if sera from the animal recognized the MAP0261c gene product or if it simply recognized the MBP affinity tag. Collectively, these data suggest that the 19-kDa protein is detectable and immunogenic in cattle with Johne's disease.

### IFN-γ responses of infected cattle

As an indicator of the cell-mediated responses of infected cattle to the 19-kDa protein, whole blood containing PBMCs from 9 control and 21 infected cows was stimulated with *M. avium *subsp. *paratuberculosis *sonicated whole cell lysate (WCL), MBP, or MBP-19 kDa and IFN-γ production was assayed by ELISA. These cattle were selected from a larger group because they showed no IFN-γ stimulation in response to MBP alone. IFN-γ production in response to WCL stimulation allowed for the segregation of infected cattle into three groups: suspect, positive, and high positive. After subtracting the IFN-γ responses of non-stimulated cells, suspect animals had less than 0.1 absorbance units of IFN-γ production, positive animals had 0.1 – 0.3 absorbance units of IFN-γ production, and high positive animals had more than 0.3 absorbance units of IFN-γ production. IFN-γ responses by blood mononuclear cells from infected cattle exceeded responses from control cattle for both the WCL and MBP-19 kDa (Table 1. Significant differences (P < 0.05) were found between control and high positive groups for both WCL and MBP-19 kDa protein stimulation. However, direct comparisons of the two antigen preps using mononuclear cells from the same animal clearly showed the WCL was a stronger stimulator of IFN-γ production (Table 1).

## Discussion

A majority of the research on individual mycobacterial proteins has been performed in *M. tuberculosis*, whereas little is known about the *M. avium *subsp. *paratuberculosis *proteome. Indeed, all currently available antigen-based diagnostic tests for Johne's disease use an undefined mixture of proteins, such as purified protein derivative (PPD) or WCL, which may not be specific for *M. avium *subsp. *paratuberculosis*. Recent completion of the *M. avium *subsp. *paratuberculosis *genome has already advanced efforts to identify novel antigens [26,34]. Furthermore, the genome will be a critical resource in proteomic studies directed at defining the proteins present in mixtures such as johnin PPD. The present study was performed in order to characterize the *M. avium *subsp. *paratuberculosis *19-kDa protein, as well as assess its immunostimulatory capabilities in cattle.

In this study, we show that the 19-kDa protein of *M. avium *subsp. *paratuberculosis *can be readily overexpressed as a fusion protein in *E. coli*. This is not true for many other proteins encoded by *M. avium *subsp. *paratuberculosis *[34] and suggests the putative lipoprotein is not toxic to *E. coli*. Previous studies have suggested the *M. tuberculosis *19-kDa protein undergoes posttranslational modification by the addition of fatty acids to form a lipoprotein [35]. Although not demonstrated directly by our studies, it is possible that the 19-kDa was not posttranslationally modified by the heterologous *E. coli *host. It is unclear whether posttranslational modification of this protein would affect its immunological activity. The recombinant antigen was detected by sera from cattle with Johne's disease; however, it was not as strong a stimulator of proliferative T-cell responses as has been reported for its counterpart in *M. tuberculosis *[36]. Furthermore, the 19-kDa protein from *M. tuberculosis *was shown to induce both cellular and humoral immune responses from mice and humans [11,14]. These studies, combined with the present study, may suggest that acylation is more important in cell-mediated immune responses than in the humoral immune response.

It is generally accepted that cellular and humoral immune responses of *M. avium *subsp. *paratuberculosis*-infected cattle are biphasic, with IFN-γ responses detected early and antibody responses detected late in infection. However, evidence suggests that an unknown *M. avium *subsp. *paratuberculosis *protein can be detected by antibodies from cattle just 3 weeks after infection [37]. As a measure of cellular immune responses, blood mononuclear cells from infected cattle were stimulated with both the recombinant 19-kDa protein and a whole-cell sonicated lysate of *M. avium *subsp. *paratuberculosis *(WCL), and IFN-γ production was measured. Results from this study suggest that while the 19-kDa protein is a stimulator of IFN-γ production, it is not as potent when compared to WCL. Additionally, we found that a majority (9 of 14) of infected cattle produced antibodies to the 19-kDa protein, as determined by immunoblot analysis. By comparison, sera from the majority of control, non-infected cattle (8 of 9) did not react to the 19-kDa protein. The single non-infected cow that did show reactivity to the MAP0261c gene product may be attributed to exposure of environmental mycobacteria such as *M. avium *subsp. *avium*, which has a similar protein (Figure 1). Sera from four clinical cows reacted to the MBP protein, but this reactivity was extremely weak. MBP is found in environmental *E. coli *and likely accounts for reactivity seen in some cattle. In order to avoid potential cross-reactivity with MBP, we attempted to cleave the MBP portion from the MBP-19 kDa fusion protein, but cleavage was not 100% efficient (data not shown).

The *M. tuberculosis *19-kDa protein was reported to contain a 21 amino acid N-terminal signal peptide [22], however our SignalP analysis for *M. avium *subsp. *paratuberculosis *identified a putative 34 amino acid N-terminal signal sequence. Furthermore, the computer algorithm PSORTb predicts a putative signal sequence, but it cannot determine if the protein is actually secreted. Antibodies will be produced against the recombinant MBP-19 kDa protein to determine if the protein is secreted by *M. avium *subsp. *paratuberculosis*.

## Conclusions

The results from this study show that the recombinant 19-kDa protein stimulates a weak host immune response in infected cattle. The 19-kDa protein may be used in conjunction with other antigens from *M. avium *subsp. *paratuberculosis *to identify infected cattle but should not be used as a "stand alone antigen" in new diagnostic assays.

## Methods

### Bacterial strains and culture conditions

*M. avium *subsp. *paratuberculosis *strain 19698-1974 (originally isolated in 1974 from a clinical cow housed at the National Animal Disease Center, Ames, IA) was grown in Middlebrook 7H9 liquid media (pH 6.0) supplemented with 10% oleic acid albumin dextrose complex (Becton Dickinson Microbiology, Sparks, MD), 0.05% Tween 80 (Becton Dickinson Microbiology), and 2 mg/ml mycobactin J (Allied Monitor Inc., Fayette, MO). *M. avium *subsp. *paratuberculosis *cultures were grown to log phase at an optical density 540 nm (OD_540_) of 0.4, at 37°C without shaking. *Escherichia coli *DH5α cells were routinely grown in Luria-Bertani (LB) broth or LB agar plates at 37°C supplemented with ampicillin (100 μg/ml) for selection.

### Cattle

The Johne's Disease Research Project at the National Animal Disease Center has a repository of sera from cattle that were euthanized with clinical signs of Johne's disease, which included shedding, weight loss and diarrhea. Fecal samples from each of these clinical cattle were found to contain more than 100 CFU per gram of feces as determined by colony counts on Herrold's egg yolk media (HEYM) agar slants by standard culture methods [38]. Sera from 14 clinical cattle were selected for immunoblot analysis. The National Animal Disease Center also maintains a small herd of non-infected cattle as well as a herd of cattle naturally infected with *M. avium *subsp. *paratuberculosis*. The animals used in IFN-γ experiments were placed in four groups consisting of 9 non-infected healthy cows, 9 suspect subclinical cows (IFN-γ responses < 0.1), 6 positive subclinical cows (IFN-γ responses 0.1 – 0.3), and 6 high-positive cows (IFN-γ responses > 0.3). The non-infected control cows were characterized by repeated negative fecal cultures performed quarterly over a 3- to 5-year period. In addition, these animals were negative on all immunological assays (i.e. ELISA and IFN-γ production) performed during that period. Subclinical cattle (suspect, positive, and high-positive) were characterized by shedding less than 10 CFU/g of feces and were intermittently positive by IFN-γ assays (response > 0.1) performed quarterly over a 3- to 5-year period. The institutional Animal Care and Use Committee approved all animal procedures described in this study.

### Comparison of the mycobacterial 19 kDa coding region

The nucleotide sequences for the 19 kDa coding regions from *M. tuberculosis*, *M. bovis*, *M. avium *and *M. intracellulare *were obtained from the NCBI nucleotide sequence database. The sequences were assembled and compared using MegAlign software (DNASTAR, Inc., Madison, WI).

### Cloning and expression of the *M. avium *subsp. *paratuberculosis *19-kDa gene

A maltose binding protein (MBP) fusion of the *M. avium *subsp. *paratuberculosis *19-kDa sequence (MBP-19 kDa) was constructed using the pMAL-c2 vector (New England Biolabs, Beverly, MA). To amplify the 19-kDa coding region from *M. avium *subsp. *paratuberculosis*, primers were designed directly from the MAP0261c sequence. MAP0261c was amplified with Expand High Fidelity PCR system using the primers 19-kDa-pMal5' (5'-GCGCCAGCTGACGATCGCGGTCGCGGGCGCGGC-3') and 19-kDa-pMal3' (5'-GCGCAAGCTTCAGGTCACATCGATCTCGAAC-3'). The 19-kDa-pMal5' and 19-kDa-pMal3' primers contained *Pvu *II and *Hin*d III restriction sites (underlined) for cloning, respectively. This primer set amplified the 19-kDa coding sequence minus the N-terminal 4 amino acids and the C-terminal 3 amino acids. The pMal-c2 vector was digested with *Xmn *I and *Hin*d III and the PCR amplicon was digested with *Pvu *II and *Hin*d III. The two products were ligated overnight at 12°C with T4 DNA ligase (Life Technologies Inc., Rockville, MD), which resulted in an in-frame fusion between the vector-encoded *malE *gene and a majority of the 19-kDa gene. The resulting recombinant plasmid, designated pMal-19-kDa, was transformed into *E. coli *DH5α competent cells. Recombinant clones were selected by plating on LB-ampicillin plates overnight at 37°C. Individual clones were picked and inserts were confirmed by DNA sequencing. The resulting fusion protein was overexpressed and purified by maltose affinity chromatography using amylose resin (New England Biolabs). A detailed expression and purification protocol has been published previously [39]. Expression and purification of the MBP-19 kDa fusion protein was monitored by sodium dodecyl sulfate-polyacrylamide gel electrophoresis (SDS-PAGE) gels either stained with GelCode Blue (Pierce Biotechnology Inc., Rockford, IL) or checked by immunoblot analysis with a monoclonal antibody against MBP developed at the National Animal Disease Center. Expression and purification of the MBP alone (without 19-kDa) was previously described [26].

### SDS-PAGE and immunoblotting

*E. coli *lysates expressing MBP-19 kDa were prepared as previously described [40]. SDS-PAGE was performed using 12% (wt/vol) polyacrylamide gels. Proteins were electrophoretically transferred onto nitrocellulose membranes (Schleicher and Schuell, Keene, NH) using the Trans Blot Cell (Bio-Rad Laboratories, Hercules, CA) in sodium phosphate buffer (25 mM; pH7.8) at 0.9 amps for 90 minutes. After transfer, the blots were blocked overnight with PBS plus 2% bovine serum albumin (BSA) and 0.1% Tween 20 (PBS-BSA). For immunoblots, serum from cattle with Johne's disease was diluted 1:500 in PBS-BSA. Sera were incubated on the blots at room temperature for 2 hours. After 3 washes in PBS plus 0.1% Tween 20, blots were incubated for 1.5 hours in anti-goat peroxidase-conjugated secondary antibody diluted 1:20,000 in PBS-BSA (Pierce Biotechnology Inc.). After secondary antibody incubation, the blots were washed 3 times as described above and were developed for chemiluminescent detection using SuperSignal detection reagents (Pierce Biotechnology Inc.).

### IFN-γ assays

Blood was collected from the jugular vein of subclinically-infected cattle into sodium heparin vacutainer blood collection tubes. One ml aliquots of whole blood from each animal were plated into 4 wells of 24-well culture plates and cultured alone (non-stimulated) or with 10 μg/ml of *M. avium *subsp. *paratuberculosis *sonicate (WCL), 10 μg/ml of MBP, or 10 μg/ml of MBP-19 kDa. The WCL was prepared by sonication of bacilli and centrifugation exactly as described previously [37]. Blood-antigen mixtures were incubated for 18 hours at 39°C in a 5% CO_2 _humidified atmosphere. Plates containing blood-antigen samples were centrifuged at 500 × g for 15 minutes and the plasma was harvested from each well. Plasma samples were frozen at -20°C until being analyzed for IFN-γ concentration by enzyme-linked immunosorbent assay (ELISA) using a commercial kit (Bovigam, BioCor, Omaha, NE) as recommended by the manufacturer. Samples were analyzed in duplicate and were determined to be positive for IFN-γ production if the absorbance of the stimulated sample (WCL, MBP, MBP-19 kDa) was 0.1 units greater than the absorbance of the nonstimulated well for that animal. This classification of IFN-γ positive samples has been previously reported by our laboratory as well as others [41,42].

### Statistical analysis

ANOVA and unpaired t tests were performed to analyze the IFN-γ stimulation data. Analyses were performed to compare average stimulation of control, non-infected cattle to the infected cattle groups (suspect, positive, high positive). Differences were considered significant when P < 0.05.

## Authors' contributions

JFH carried out all the experiments and drafted the manuscript as part of his PhD dissertation. JRS provided advice, participated in its design and coordination and helped edit the manuscript. JPB conceived of the study, participated in its design and helped to draft the manuscript.

## Figures and Tables

**Table 1:** IFN-γ production by PBMCs in response to WCL and MBP-19 kDa stimulation.

	**CONTROL WCL**	**CONTROL 19 KDA**	**SUSPECT WCL**	**SUSPECT 19 KDA**	**POSITIVE WCL**	**POSITIVE 19 KDA**	**HIGH POS WCL**	**HIGH POS 19 KDA**
	0	0	0.0294	0.0017	0.1058	0.0056	1.2407	0.9212
	0.0623	0.0513	0.0980	0	0.1013	0.7737	1.0966	1.125
	0.0072	0.0280	0.0412	0.1036	0.1323	0	0.3719	0.0124
	0	0	0.0051	0.0286	0.1909	0.0290	0.3348	0.0925
	0	0.0429	0.0259	0.0735	0.1623	0.0245	1.1866	0.7230
	0.0560	0.0076	0	0	0.1974	0.0299	2.5995	0.0536
	0.0561	0.0183	0.0348	0				
	0.0066	0.0285	0.0203	0.0071				
	0.0293	0.0236	0.0268	0.0002				
**Mean**	**0.0242**	**0.0222**	**0.0313**	**0.0239**	**0.1483**	**0.1438**	**1.1384**	**0.4880**
